# First time identification of *Pandoraea sputorum* from a patient with cystic fibrosis in Argentina: a case report

**DOI:** 10.1186/s12890-017-0373-y

**Published:** 2017-02-07

**Authors:** Pablo F. Martina, Mónica Martínez, Guillermo Frada, Florencia Alvarez, Lorena Leguizamón, Claudia Prieto, Carolina Barrias, Marisa Bettiol, Antonio Lagares, Alejandra Bosch, Julián Ferreras, Martha Von Specht

**Affiliations:** 1 Instituto de Biología Subtropical (IBS), CONICET-UNaM, Misiones, Argentina; 2 Hospital Pediátrico Dr F. Barreyro, Posadas, Misiones Argentina; 3 Instituto de Biotecnología y Biología Molecular (IBBM) - CONICET/UNLP, La Plata, Argentina; 4 Hospital de Niños Sor María Ludovica, La Plata, Argentina; 5 Centro de Investigación y Desarrollo en Fermentaciones Industriales (CINDEFI) - CONICET/UNLP, La Plata, Argentina; 6 Facultad de Ciencias Exactas, Químicas y Naturales, Universidad Nacional de Misiones, Posadas, Argentina

**Keywords:** *Pandoraea sputorum*, Cystic fibrosis, First report, MALDI-TOF MS, Argentina

## Abstract

**Background:**

*Pandoraea* species are considered emerging pathogens in the context of cystic fibrosis (CF) and are difficult to identify by conventional biochemical methods. These multidrug resistant bacteria remain poorly understood particularly in terms of natural resistance, mechanisms of acquired resistance and impact on the prognosis of the disease and the lung function. Among them, *Pandoraea sputorum* has been previously described in few cases of CF patients from Spain, Australia, France and United States, underlining the need of more clinical data for a better knowledge of its pathogenicity. This is the first report relating to *P. sputorum* in a CF patient in Argentina.

**Case presentation:**

*Pandoraea sputorum* was identified in a nine-year-old cystic fibrosis patient from Argentina, after treatment failure during an exacerbation. The isolates were successfully identified by combining molecular techniques based on 16S rRNA sequencing and mass spectrometry (MS) methods, after reassessing previous misidentified isolates by conventional methods. After first isolation of *P. sputorum*, patient’s clinical condition worsened but later improved after a change in the treatment. Although isolates showed susceptibility to trimethoprim–sulfamethoxazole and imipenem, in our case, the antibiotic treatment failed in the eradication of *P. sputorum*.

**Conclusions:**

All combined data showed a chronic colonization with *P. sputorum* associated to a deterioration of lung function. We noted that the presence of *P. sputorum* can be underestimated in CF patients and MALDI-TOF MS appears to be a promising means of accurate identification of *Pandoraea* species.

## Background

In cystic fibrosis (CF) patients, the main cause of morbidity and mortality is the decline in the pulmonary function subsequent to pathogenic colonization with non-fermenting Gram negative bacteria (NFGNB) [1]. Nevertheless, diverse bacterial species exhibit distinct degrees of pathogenicity and sensitivity patterns, requiring different clinical managements [2]. Regularly, a series of opportunistic pathogens colonizes the CF lung throughout life, often culminating in chronic infection with *Pseudomonas aeruginosa* and, less frequently, with *Burkholderia cepacia* complex (Bcc) organisms. Other opportunistic Gram-negative bacteria are also encountered. They include various Enterobacteriaceae, *Stenotrophomonas maltophilia*, *Achromobacter xylosoxidans* [1, 3] and species that are only occasionally associated with human infections apart from CF, such as *Cupriavidus*, *Ralstonia*, and *Pandoraea* species. Some of these species have been associated with poor outcomes in CF, and the role that others may play in disease progression remains uncertain [2]. Bacteria from the *Pandoraea* genus (*P. pulmonicula*, *P. pnomenusa*, *P. apista*, *P. norimbergensis and P. sputorum*) are NFGNB that are being considered as emerging multi-drug resistant pathogens in the context of CF [4, 5]. As they are still poorly known, particularly in terms of mechanisms of natural or acquired resistance and of their impact on lung function and the prognosis of the disease [5], more information is needed it to evaluate their pathogenic role. Conventional microbiology methods remain problematic for the accurate identification of *Pandorea* species and misidentification with bacteria of the genus *Burkholderia* or *Ralstonia* has been reported [6–8]. Moreover, isolates from CF patients with persistent pathogenic colonization often lose their characteristic phenotypes or growth conditions [9]. Therefore, descriptions of infections due to *Pandoraea* species in CF patients could be underestimated. The availability of MALDI-TOF MS in clinical microbiology diagnosis allows a quick and better identification of bacterial species, especially *Pandoraea* species [5, 8, 10–12]. In this study we report for the first time in Argentina, a lung colonization with *Pandoraea sputorum*, in a young CF patient who did not respond to antibacterial empirical treatment. Identification was possible after reassessing historical identification results obtained by conventional methods, using MALDI-TOF MS and sequencing of 16S rRNA gene.

## Case presentation

**Fig. 1:**
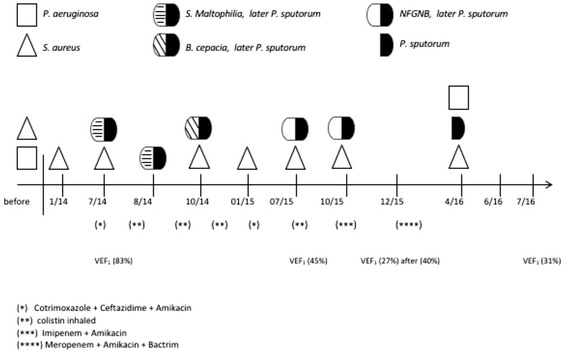
Time course outlines antibiotics treatment, lung function and culture results

A 9-year-old boy who was diagnosed with CF at 29 months (heterozygous genotype F508del; sweat test positive: Na 74 mEq/l and Cl de 63 mEq/l.), was transferred to our CF unit with a 5 years history of chronic bronchopulmonary colonization with *Pseudomonas aeruginosa* and *Staphylococcus aureus*. When the first microbiological cultures (always sputum samples) were performed in our laboratory in July 2014, *P. sputorum* was detected together with *S. aureus*, but was initially reported as *S. maltophilia* (Fig. 1). Previously, his pulmonary function test revealed a forced expiratory volume in 1 s (FEV_1_) of 83%, and the preceding respiratory exacerbations were treated empirically with ceftazidime, amikacin and with aerosolized tobramycin. From the time of the first isolation of *P. sputorum* and during the next 2 months, his clinical condition worsened and exacerbations with increased cough and secretions were observed. The patient was treated with trimethoprim-sulfamethoxazole (4/20 mg/kg/day) plus ciprofloxacin (15 mg/kg/day) and with aerosolized colistin (2 million units twice daily), however, his lung function declined (FEV_1_ 45%). In June 2015, the patient showed exacerbations with a dramatic deterioration of his pulmonary function (FEV_1_ 27%). A combined therapy for three-weeks of imipenem (80 mg/kg/day), amikacin (40 mg/kg/day), and inhaled colistin was initiated. Consequently, his clinical condition improved with a significant reduction in sputum volume and cough (FEV_1_ 40%). Five months later, his clinical condition was stable. In April 2016, a new respiratory sample was positive for *P. sputorum*, confirmed by MALDI-TOF MS, together with with *S. aureus* and *P. aeruginosa*.

**Fig. 2:**
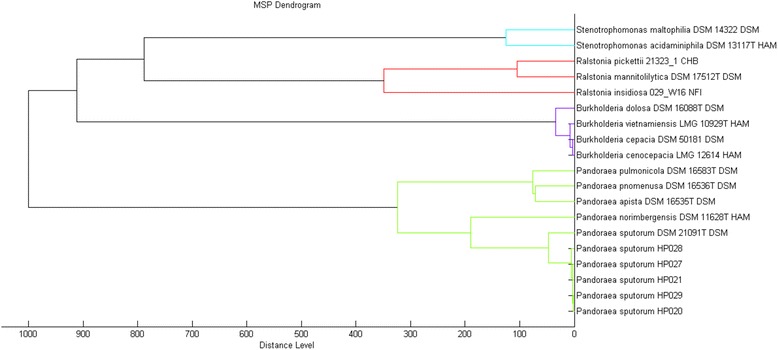
Cluster analysis of MALDI-TOF MS spectra of *Pandoraea sputorum* isolates and 14 reference strains of related species (MALDI Biotyper 3.1 database). A standard MainSpectrum was created to clinical isolate *P. sputorum* HP020. Isolates from the same patient are represented in MSPs strains 0020, 0021, 0027, 0028 and 0029. Distance is displayed in relative units

**Fig. 3:**
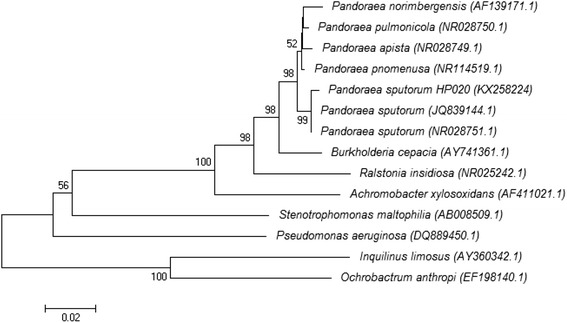
Phylogenetic tree based on the 16S rDNA sequence of *Pandoraea sputorum* HP020 (KX258224) strain. The tree was constructed using a neighbor-joining method, and 1000 bootstrap by using MEGA6 software as previously described [22]. Values above the lines are bootstrap values expressed as percentages

Our laboratory records indicated that sputum cultures during patient’s exacerbations and/or routine follow-up visits until July 2015, showed colonies of NFGNB without any clear phenotypic differences and *S. aureus*. Conventional identification of purple colonies recovered on *B. cepacia* complex selective agar plates (BCSA; Britania S.A, Argentina) was performed to some NFGNB colonies. We used a combination of phenotypic tests [13] like oxidase activity, DNase, carbohydrate oxidation-fermentation, lysine and arginine decarboxylase, and motility tests. After these tests, isolates were reported as NFGNB (n = 2), *S. maltophilia* (n = 2), *B. cepacia* complex (n = 1) and *S. aureus* (n = *7*). Later, one of the Gram-negative isolates was identified as *Bordetella hinzii* with a high identification confidence level (99% probability) using VITEK2 automatic system (bioMérieux, France). Due to the inaccuracy of biochemical tests, we decided to reassess the identification of all NFGNB isolates (n = 5) by Matrix Assisted Laser Desroption Ionisation-Time of flight (MALDI-TOF) mass spectrometry (MS), using an Ultraflex MALDI TOF/TOF instrument and the MALDI Biotyper 3.1 software (Bruker Daltonics, Bremen, Germany). The five isolates were identified as *Pandorea sputorum* at species-level (score >2). Hierarchical cluster analysis based on MALDI-TOF MS spectra comparison clustered together, clinical isolates and the reference strain *P. sputorum* DSM 21091^T^, while *P. apista*, *P. pnomenusa*, *P. pulmonicola* and *P. norimbergensis* were in a separate branch (Fig. 2). In addition, genetic relatedness was determined by fingerprinting analysis like BOX-PCR (Interspersed repetitive element) and ERIC-PCR (enterobacterial repetitive intergenic consensus) [14], revealing indistinguishable patterns among all our clinical isolates (data not shown). Finally, a subsequent full characterization through PCR and sequencing of 16S rRNA gene was performed using the corresponding universal primers (27 F and 1492R), previously published [15]. Sequence analysis of a 1400-bp fragment of the 16S rRNA gene of one of patient’s isolate (*P. sputorum* HP020) was performed. The corresponding sequence (GenBank accession KX258224) showed 99% of similarity with *P. sputorum* DSM21091^T^ (GenBank accession CP010431), which confirmed the results obtained by MALDI-TOF MS analysis. The phylogenetic relationship among them was investigated by using MEGA 6.0 software (http://www.megasoftware.net) (Fig. 3).

Antibiotic susceptibility testing was performed using a disk diffusion method according to the recommendations of the committee of CLSI [16]. Susceptibility patterns were also identical for all isolates. In the absence of interpretive susceptibility criteria for strains of *Pandoraea*, results were interpreted using CLSI criteria for “other non-*Enterobacteriaceae*” isolates. The microorganism was resistant to amikacin, ceftazidime, cefepime, meropenem, gentamicin, aztreonam and colistin and susceptible to imipenem and trimethoprim-sulfamethoxazole. Minimum inhibitory concentrations (MIC, mg/L) were estimated by Vitek2 Compac® for amikacin (≥64), ceftazidime (≥64), cefepime (≥64), ciprofloxacine (≥4), colistin (≥16), meropenem (≥16), gentamicin (≥64), piperacillin-tazobactam (≥128), imipenem (≤0,25) and trimethoprim-sulfamethoxazole (TMS, ≤ 1/20), which confirms the susceptibility profile.

## Discussion and Conclusions

In the last decade, many emerging multidrug resistant pathogens have been described in CF patients including new species of the *Burkholderia cepacia* complex, *Brevundimonas diminuta*, *Inquilinus limosus*, *Acetobacter indonesiensis*, *Achromobacter xylosoxidans*, *Ochrobactrum anthropi*, and bacteria of the genus *Pandoraea* [17, 18]. The pathogenicity of *Pandoraea* species remains controversial. These bacteria have been occasionally recovered from the lung transplant and/or respiratory tract of CF patients, as well as from blood cultures in non-CF patients and environmental samples [2, 4, 8, 19]. In our review of the bibliography, we found few cases of CF patients chronically colonized with *P. sputorum*: one case from Australia [8], two cases from Spain [10, 11], and one case from France [5]. Interestingly, in our CF patient, *S. aureus* and *P. aeruginosa* were eventually isolated from respiratory tract cultures together with *P. sputorum*. This situation makes more difficult to know the real contribution of *P. sputorum* to the clinical condition of our patient. In this regard, intermittent or persistent colonization with *Pandoraea* species, that sometimes coincides with a deterioration in lung function, has been reported in addition to clinical evidence of invasive potential [8, 10, 11, 18]. Furthermore, besides the inability of *Pandoraea* species to produce a biofilm in the respiratory tract [20], it remains still unclear the role of different virulence factors, mechanisms of pathogenicity, the possibility of transmission between patients, and their role in lung damage in CF patients. This situation emphasizes the importance of having more data about clinical cases. The *Pandoraea* genus is multidrug resistant and treatment may be problematic. In our case, *P. sputorum* was only susceptible to imipenem and TMS, with a low MIC for imipenem (≤0,25 mg/L), and although the treatment failed to eradicate the bacteria, the clinical condition improved. This pattern of being resistant to carbapenem and meropenem but sensitive to imipenem, appears to be unique to most *Pandoraea* genus [5, 21], and it can be a practical guideline in the earliest identification steps.

It is documented, when applying only conventional identification phenotypic methods, the microbiology laboratory commonly misidentifies this pathogen as *Ralstonia*, *Stenotrophomonas* or *Burkholderia* species [6–8]. It has been also noted the limitations of the sequences of 16S rRNA and *gyrB* genes for differentiating the *Pandoraea* species [8, 22]. The accuracy and usefulness of MALDI-TOF as a routine technique for rapid identification of bacterium was demostrated. Nevertheless, one factor limiting the use of MALDITOF MS is the scant reference data sets for microorganisms that are infrequently isolated from clinical specimens [10]. Due to these limitations, a polyphasic approach for an accurate identification is recommended. In our CF patient, all isolates were identified as *P. sputorum*, combining 16S rRNA PCR and sequencing, and a MALDI-TOF MS proteomic platform. Lastly, to determine persistence over time of the same *P. sputorum* isolate, as generally occurs with *P. aeruginosa* and Bcc in CF patients, a molecular epidemiological analysis was performed [2, 23]. BOX-PCR and ERIC-PCR showed indistinguishable patterns among all isolates of *P. sputorum* (includeding last isolate collected in April 2016), indicating a possible chronic colonization, which is defined here as three positive cultures of the same strain isolated within a 6-month period.

In conclusion, we noted that the presence of *P. sputorum* can be underestimated in CF patients when using conventional biochemical identification methods. We also showed that *P. sputorum* may have a potential role in pulmonary function deterioration when chronically colonized CF patients, emphasizing the need to implement control policies to protect CF community. This is particularly relevant, as in our case, the treatment failed despite sensitivity to imipenem and trimethoprim–sulfamethoxazole. Finally, MALDI-TOF MS appears to be an excellent tool for an accurate identification of organisms of this genus.

## Abbreviations

CF: Cystic fibrosis
CLSI: Clinical Laboratory Standards Institute
FEV1: Forced expiratory volume in one second
MALDI-TOF MS: Matrix-assisted laser desorption ionization-time of flight mass spectrometry
MIC: Minimum inhibitory concentration
NFGNB: Non-fermenting Gram-negative bacteria
